# Screening of *Solanum* (sections *Lycopersicon* and *Juglandifolia*) germplasm for reactions to the tomato brown rugose fruit virus (ToBRFV)

**DOI:** 10.1007/s41348-021-00535-x

**Published:** 2021-10-09

**Authors:** Ahmad Jewehan, Nida Salem, Zoltán Tóth, Pál Salamon, Zoltán Szabó

**Affiliations:** 1 Applied Plant Genomics Group, Institute of Genetics and Biotechnology, Hungarian University of Agriculture and Life Sciences, Gödöllő, 2100 Hungary; 2 Department of Plant Protection, School of Agriculture, The University of Jordan, Amman, 11942 Jordan

**Keywords:** ToBRFV, Wild tomatoes, *S. ochranthum*, Resistance, Susceptibility

## Abstract

The reaction of 636 *Solanum* (sections *Lycopersicon* and *Juglandifolia*) accessions were evaluated under greenhouse conditions after mechanical inoculation with a Jordanian isolate of the new tobamovirus tomato brown rugose fruit virus (ToBRFV). Local and systemic infections were assayed by symptoms evaluation and virus detection via biotests and RT-PCR. All cultivated tomatoes (*Solanum lycopersicum*) and the great majority of wild tomato accessions proved susceptible to ToBRFV. They showed a wide range of symptoms (mosaic, leaf deformations, mottling, shoestring, and stunting). Twenty-six accessions representing *S. lycopersicum* var. cerasiforme, *S. pimpinellifolium*, *S. habrochaites,* and *S. chilense* were tolerant. High levels of resistance have been demonstrated in three accessions of *S. ochrantum*, a close relative to wild tomatoes (member of the sect. *Juglandifolia*) not only to ToBRFV but also to the tobamoviruses, tobacco mosaic virus (TMV) and tomato mosaic virus (ToMV). After mechanical inoculation, the three tobamoviruses could be detected only in inoculated leaves in the accessions LA2160, LA2162, and LA 2166, which remained symptomless. However, two other *S. ochrantum* accessions PI 473,498 and PI 230,519 reacted unusually. They were demonstrated highly resistant to TMV and ToMV, but proved transiently susceptible to ToBRFV showing mild systemic mosaic followed by total recovery from symptoms and the virus.

**Supplementary Information:**

The online version contains supplementary material available at 10.1007/s41348-021-00535-x.

## Introduction

Tomato (*Solanum lycopersicum*) is one of the most important vegetables produced and consumed worldwide (Heuvelink 2018). Similar to other solanaceous plants, pathogens often cause heavy losses of fruit quality and yield of the crop. Tomato is susceptible to a wide range of viruses, out of which tobacco mosaic virus (TMV) and tomato mosaic virus (ToMV) have been ranked as the most important tomato pathogens (Jones et al. 2016). TMV and ToMV are related viruses belonging to the genus *Tobamovirus*, family *Virgaviridae* (Adams et al. 2009). Typically, they have rod-shaped particles of 300 × 18 nm in size, which encapsidate the genomic single-stranded positive-sense RNA. The viral particles are very stable, highly infectious, and can be spread easily via mechanical transmissions through wounds caused by workers or pollinator insects (Okada et al. 2000; Levitzky et al. 2019). Moreover, TMV and ToMV can also be transmitted by tomato seeds, one of the most important sources of infection (Dombrovsky and Smith 2017). All of these viral properties make the control of tobamovirus infections difficult.

Till now, breeding and use of resistant *S. lycopersicum* cultivars proved to be the most effective strategy to control tobamoviruses. Three tobamovirus resistance genes [*Tm-1, Tm-2*, and *Tm-2*^*2*^ (*Tm-2*^*a*^*)*] have been transferred into *S. lycopersicum* via crossing with wild tomato species*.* The *Tm-1* derived from *S. habrochaites* (PI 126,445) is an incompletely dominant gene that suppresses virus replication (Holmes 1954; Pelham 1972; Fraser et al. 1980). The *Tm-2* and *Tm-2*^*2*^ alleles were introgressed from *S. peruvianum* (PI 126,926, PI 128,650), conferring complete dominant resistance based on the hypersensitive reaction of the host plant (Laterrot and Pecaut 1969; Alexander 1963; Schroeder et al. 1967; Pfitzner 2006)**.**

Resistant breaking strains of TMV or ToMV have been detected for decades (Betti et al. 1997; Calder and Palukaitis 1992), but these strains did not spread widely in tomato crops until now. However, a new tobamovirus first isolated in Jordan and named as Tomato brown rugose fruit virus (ToBRFV) (Salem et al. 2016) caused a “pandemic alert” in Europe (EPPO Global Database 2016).

ToBRFV is a high-risk pathogen due to its rapid distribution across several continents (Africa, Asia, and North America) and its ability to overcome the resistance genes *Tm-2* and *Tm-2*^*2*^ introgressed into tomato during the past decades (Luria et al. 2017). The virus infection can occur by seed transmission as primary inoculum and pollen transmission of bumblebee (Bombus terrestris) (Dombrovsky and Smith 2017; Levitzky et al. 2019; Davino et al. 2020; Salem et al. 2021). Symptoms caused by ToBRFV vary greatly depending on the genotype, age, and environment of the infected tomato. Foliar symptoms usually appear as chlorosis, mosaic patterns, and mottling, occasionally accompanied with leaf narrowing. Fruits of diseased plants show yellow or brown wrinkled (rugose) patches rendering them unmarketable (Salem et al. 2016; Luria et al. 2017). Besides tomato, ToBRFV is able to infect sweet pepper (*Capsicum annuum*) (Salem et al. 2020; Panno et al. 2020). Because the resistance genes are not active to ToBRFV, there is an urgent demand to find new sources of resistance. The present study aimed to screen for the susceptibility and resistance of a wide range of wild tomatoes and some *Solanum* relatives to ToBRFV.

## Materials and methods

### Plant materials

A total of 636 plant accessions belonging to different *Solanum* (sections *Lycopersicon* and *Juglandifolia*) species such as *S. arcanum* (9); *S. cheesmaniae* (21); *S. chilense* (99); *S. chmielewskii* (10); *S. corneliomulleri* (26); *S. galapagense* (11); *S. habrochaites* (22); *S. huaylasense* (9); *S. juglandifolium* (3); *S. lycopersicum* (81); ‏*S. neoricki* (16); *S. ochranthum* (5); *S. pennellii* (18); *S. peruvianum* (43); *S. pimpinellifolium* (256); and *S. sitiens* (7) were investigated. The following accessions were used as a control: *S. habrochaites* (PI 126,445; original source of the *Tm-1* gene), *S. peruvianum* (PI 126,926; source of the *Tm-2* gene, and PI 128,650; source of the *Tm-2*^*2*^ gene), *S. lycopersicum* (LA1221; carrying the introgressed *Tm-2*^*2*^ gene), and the susceptible cultivar *S. lycopersicum* (‘Ceglédi’; +*/*+). The seeds of *Solanum* species were kindly supplied by the Tomato Genetic Resources Centre (University of California, Davis), United States Department of Agriculture Agricultural Research Service (Beltsville, Maryland) and MATE (Hungarian University of Agriculture and Life Sciences). The seeds were sown in fertilized Klasmann Traysubstrate soil, and potted plants were grown in an insect-proofed glasshouse at 24 ± 2 °C temperature, 14/10 h photoperiod, and 50–70% relative humidity.

### Virus isolates and plant inoculation

A Jordanian ToBRFV isolate (GenBank acc.no. MZ323110) was employed in the present work. Tobacco mosaic virus (TMV-U1) and tomato mosaic virus (ToMV-DH) isolates were received from the virus collection of MATE (Hungarian University of Agriculture and Life Sciences) kindly provided by Pál Salamon. ToBRFV, TMV, and ToMV were transmitted through a single local lesion in *Nicotiana glutinosa* and propagated on *N. tabacum* cv. Samsun. Inocula were prepared by grinding infected tobacco leaves in sterile 0.01 M phosphate buffer pH 7.0 (1:5 w/v). The sap was then filtered through a cheesecloth, and the extract was stored in aliquots at − 20 C^0^ for inoculation through this work.

For mechanical transmission, virus inoculum was rubbed onto carborundum-dusted lower leaves of young tomato and tobacco test plants. At least 3–10 plants from each accession were inoculated at 3–4 true leaf stage. Local and systemic symptoms were evaluated 3–4 weeks post-inoculation (wpi). In each inoculation experiment, the infectivity of the inocula was assayed using *N. glutinosa* and/or *N. tabacum* cv. Xanthi-nc as local lesion test plants. All greenhouse and laboratory experiments were carried out in quarantine conditions.

### Evaluation of disease symptoms and handling of resistant plants

The disease caused by ToBRFV was assessed in each inoculated plant 2–3 wpi, and the disease severity index (DSI) listed (Table 1) was calculated by the formula developed by (Camara et al. 2013):

$${\text{DSI}}\left( \% \right) = \mathop \sum \limits_{e = 0}^{4} \frac{{e{\text{Re}} \times 100 }}{5N}$$

where $\mathrm{DSI}$ = disease severity index; *e* = class; Re = number of plants in class (*e*); *N* = total number of plants.

**Table 1:** Symptom severity classes on top leaves of inoculated plants

Classes	Symptoms
0	No symptoms
1	Mild mosaic or mottling, followed by recovery
2	Mild mosaic or mottling with leaf deformation
3	Moderate mosaic or mottling and leaf deformation followed by rolling
4	Severe mosaic or mottling, and leaf deformity
5	Severe mosaic or mottling, leaf deformity, shoestring

Symptomless plants (class 0) confirmed as virus-free by bioassay and RT-PCR were predicted to be resistant. These plants were re-inoculated and tested for the presence of the virus again. Three weeks after the second inoculation, the symptomless plants were decapitated. Two weeks later, two lateral shoots from the decapitated plants were inoculated again and checked for the presence of the virus. Simultaneously, one non-inoculated lateral shoot of each plant was cut off, rooted in MS media, propagated in vitro and transferred to pots. Three propagated individuals were inoculated by TMV, ToMV, and ToBRFV, respectively, and assayed for the presence of tobamovirus by bioassay and RT-PCR.

### Detection of viruses

The presence or absence of viruses was demonstrated in leaf samples of inoculated plants using bioassay and reverse-transcription polymerase chain reaction (RT-PCR). Samples were taken at 2–3 wpi from newly developed top leaves. The assayed leaves were rinsed with sodium hydroxide (2%) and then with tap water to avoid virus contamination. Bioassays were carried out by rubbing indicator plants (*N. glutinosa* and *N. tabacum cv. Xanthi nc*) with leaf extract prepared from inoculated and top leaves of donor tomato plants, respectively. For RT-PCR, RNA was extracted using Promega SV Total RNA extraction kit following the manufacturer’s instructions. Extracted RNA samples were used as a template for complementary DNA (cDNA) transcription oligonucleotide specific for ToBRFV, ToMV, and TMV specific primers. Primer3 computer software (version 4.0.0) was used to design the PCR primers using the ToBRFV (KT383474), ToMV (MH507165) and TMV (FR878069) reference virus genomes. To amplify the coat protein segment, the following primers were used: for ToBRFV F-5894 (5'- GTTCCAAACACAACAAGCTAGA -3') and R-6250 (5'- AAAGTGCATCCGGTTTACAATG -3'), for ToMV F-5894 (5'- GTTTCAAACACAGCAAGCAAGA -3') and R-6250 (5'- CAGACCAACCCAGACATACTTT -3'), and for TMV F-5809 (5'- CTCCATCTCAGTTCGTGTTCTTG -3') and R-6250 (5'- CAAACCAAACCAGAAGAGCTCT -3'). PCR products were detected by electrophoresis in agarose gel (2%, in 0.5 X TBE buffer).

## Results

### Evaluation of *Solanum* accessions for the susceptibility and resistance to ToBRFV

**Fig. 1:**
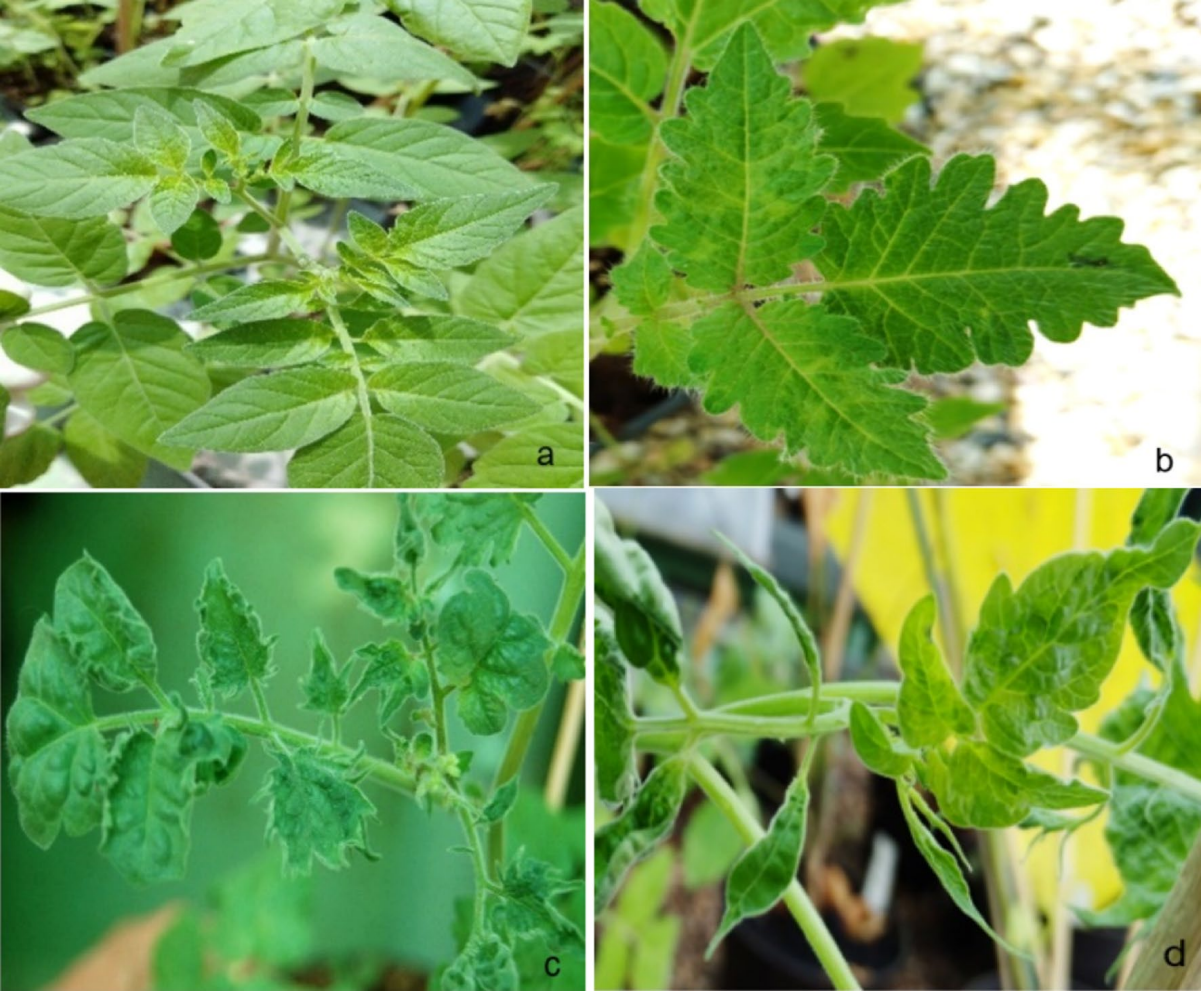
Symptoms on plants of different *Solanum* species susceptible to ToBRFV. **a** = tolerant plant, **b–d** = susceptible plants showing different severities of disease reaction (**b** = mild mosaic, **c** = severe mosaic; rolling of leaf edges, **d** = mosaic with severe leaf deformations and leaf narrowing)

**Fig. 2:**
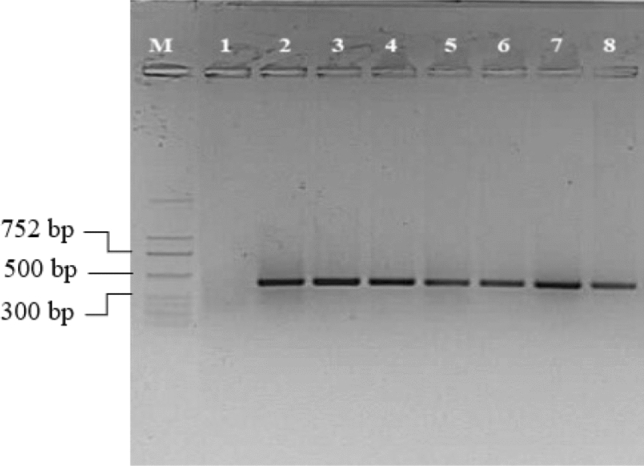
Detection of ToBRFV in plant of tolerant tomato accessions by RT-PCR. M = molecular marker, 1 = negative control, 2 = positive control, 3–6 = *S. pimpinellifolium* accession (LA1301, LA1375, LA1547, LA1924, 7–8 = *S. habrochaites* (LA1559, LA2174)

**Fig. 3:**
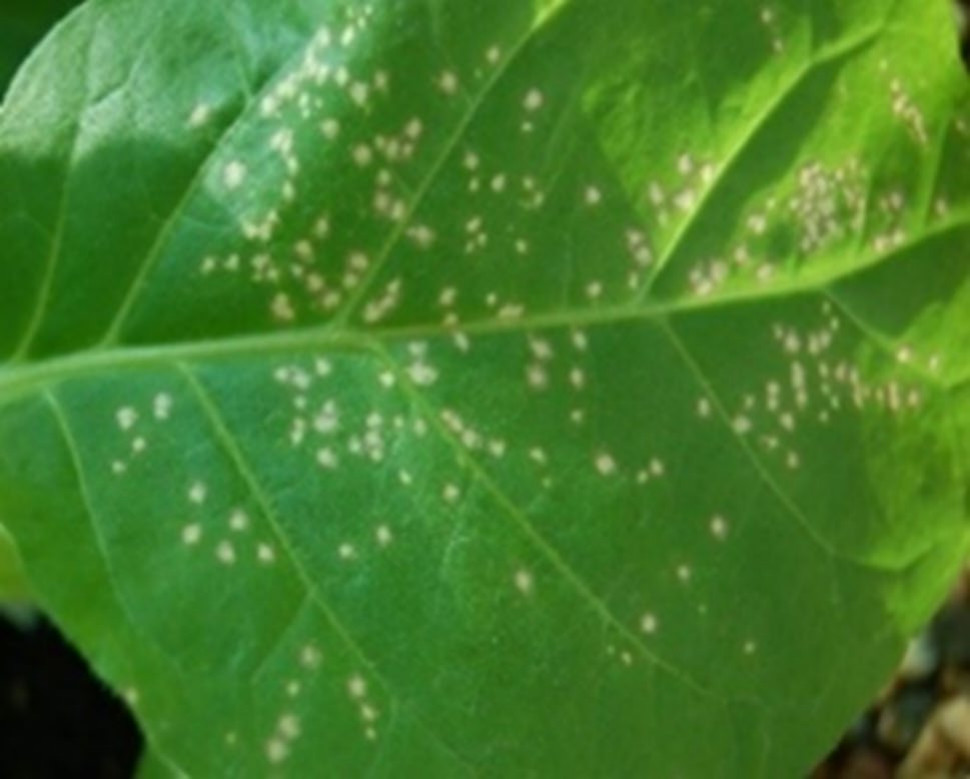
Necrotic lesions in leaf of *N. tabacum* var. Xanthi-nc plant inoculated with extract of ToBRFV infected tolerant tomato plant

Six hundred thirty-six *Solanum* accessions were inoculated with ToBRFV and evaluated for symptoms and DSI (Table 1, Supplementary 1). The control accessions, *S. peruvianum* (PI 126,926; *Tm-2*, PI 128,650; *Tm-2*^*2*^), *S. lycopersicum* (LA1221; *Tm-2*^*2*^), and *S. lycopersicum* (Ceglédi; +/+), showed DSI ranged between 80 and 100%, whereas *S. habrochaites* (PI 126,445; *Tm-1*) plants showed milder symptoms displaying a DSI of 20%. Plants of 603 wild *Solanum* accessions were susceptible and showed systemic symptoms at different severity levels (Fig. 1, Supplementary 1). Twenty-six accessions of *S. pimpinellifolium* (LA1301, LA1375, LA1547, LA1579, LA1607, LA1611, LA1612, LA1630, LA1634, LA1661, LA1670, LA1676, LA1679, LA1685, LA1728, LA1924, LA2903, LA2904, LA2982), two accessions of *S. habrochaites* (LA1559, LA2174), one accession of *S. chilense* (LA1932), and four accessions of *S. lycopersicum* var. cerasiforme (LA1456, LA2675, LA2688, LA1385) were found to be tolerant showing no symptoms at all or very mild mosaic symptoms with average disease severity between 0 and 20%. The presence of the virus in tolerant plants was confirmed by using RT-PCR and bioassays (Figs. 2 and 3).

**Fig. 4:**
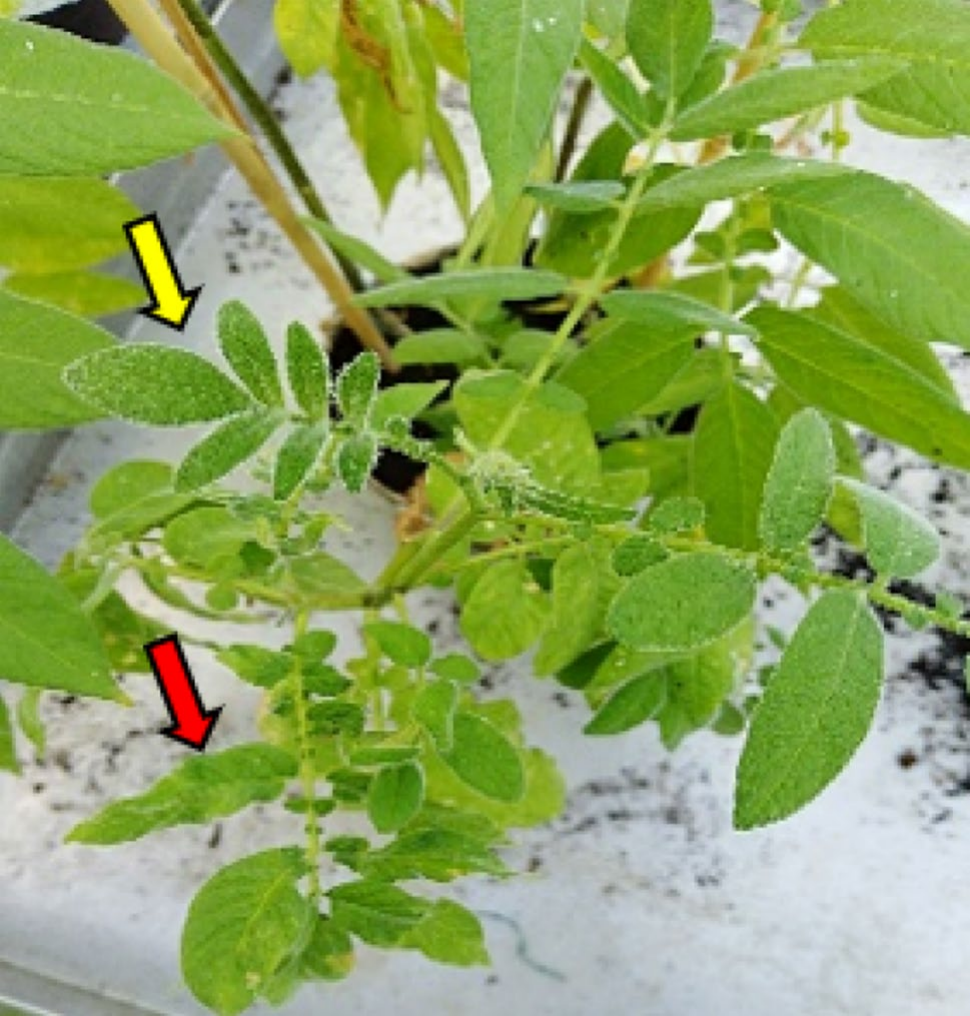
Mild mosaic symptoms (red arrow) followed by recovery (yellow arrow) on the newly developed top leaves of *S. ochranthum* PI 473,498

**Fig. 5:**
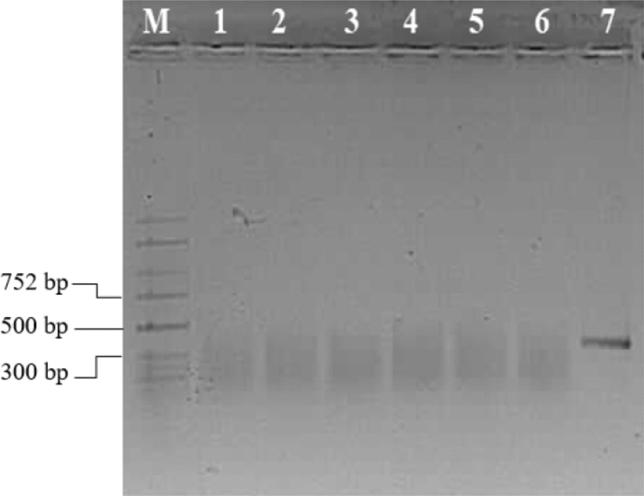
Detection of ToBRFV by RT-PCR in resistant *S. ochranthum* plants. M = molecular marker, 1 = negative control, 2 = LA2160, 3 = LA2162, 4 = LA2166, (5–6) = PI 473,498 and PI 230,519, 7 = positive control samples. Plants 5–6 showed mild mosaic after inoculation, and according to the bioassays on *N. tabacum* var. Xanthi-nc they contained virus*,* but later they recovered, and no virus could be detected in their top leaves by bioassays and RT-PCR

The inoculated and top leaves of *S. ochrantum* accessions, LA2160, LA2162 and LA2166, remained symptomless following the first, the second and the lateral shoot inoculation by ToBRFV. The presence of the virus has been detected only on inoculated leaves using bioassay. Similar responses were observed on these accessions also inoculated with TMV and ToMV, respectively. The other two *S. ochrantum* accessions PI 473,498 and PI 230,519 showed distinct responses to ToBRFV, ToMV, and TMV. They were infected only locally by TMV and ToMV but infected locally and systemically by ToBRFV. The systemic reactions of the *S. ochrantum* accessions PI 473,498 and PI 230,519 were unexpected. They initially expressed mild systemic mosaic symptoms (DSI 20%) at 15 days post-inoculation (dpi) and contained infective virus. However, they recovered from the symptoms (Fig. 4) and the virus could not be detected on their newly emerged symptomless leaves (Table 2, Fig. 5).

**Table 2:** Local and systemic reactions of *S. ochranthum* accessions to three tobamoviruses

	ToBRFV		ToMV		TMV	
*S. ochranthum* accessions	Local	Systemic	Local	Systemic	Local	Systemic
LA2160	sl (+) ^a^	sl (−) ^b^	sl (+) ^a^	sl (−) ^b^	sl (+) ^a^	sl (−) ^b^
LA2162	sl (+) ^a^	sl (−) ^b^	sl (+) ^a^	sl (−) ^b^	sl (+) ^a^	sl (−) ^b^
LA2166	sl (+) ^a^	sl (−) ^b^	sl (+)^a^	sl (−) ^b^	sl (+) ^a^	sl (−) ^b^
PI 473,498	sl (+) ^a^	mm ( +) ^a^ → sl (−) ^b^	sl (+) ^a^	sl (−) ^b^	sl (+) ^a^	sl (−) ^b^
PI 230,519	sl (+) ^a^	mm (+) ^a^ → sl (−) ^b^	sl (+) ^a^	sl (−) ^b^	sl (+) ^a^	sl (−) ^b^

sl = symptomless, mm = mild mosaic, (+) ^a^ = virus was detected by using bioassay, (−) ^b^ = virus was not detected by using bioassay and RT-PCR, →  = became symptomless on top leaves

## Discussion

Several members of the *Tobamovirus* genus like TMV and ToMV have been known for a long time as harmful pathogens of tomato. These mechanically and seed transmitted stable viruses were successfully controlled using resistant cultivars and hybrids carrying the well-known resistance genes *Tm-1,* *Tm-2,* and *Tm-2*^*2*^ (Soost 1963; Alexander 1963; Pfitzner 2006). Although some TMV and ToMV mutants have been reported to overcome the resistance conferred by these genes, they did not spread widely and no serious yield losses were reported (Betti et al. 1997; Calder and Palukaitis 1992). However, ToBRFV, a recently emerged plant virus (Salem et al. 2016), has been demonstrated to infect all the known genotypes carrying characterized resistance genes and caused worldwide panic among seed companies and tomato producers (Luria et al. 2017; Dombrovsky and Smith 2017). Resistance to this new virus has been demonstrated in some genotypes of *S. pimpinellifolium*, *S. lycopersicum* and *S. habrochaites* (Hamelink et al. 2019; Ashkenazi et al. 2020; Ykema et al. 2020), while tolerance to ToBRFV has been found in a genotype *S. lycopersicum* and *S. pimpinellifolium* (Ashkenazi et al. 2018; Zinger et al. 2021).

Studying the reaction of 636 accessions of 16 species, we found that most of them were susceptible, including the accessions of *S. arcanum*, *S. chmielewskii*, *S. huaylasense*, *S. juglandifolium*, *S. sitiens*, and *S. ochranthum* (Supplementary 1). To the best of our knowledge, the last-mentioned six species have never been studied as hosts or non-host of ToBRFV, so they can be indicated as new experimental hosts of this virus.

Several accessions were found to be tolerant (listed in Supplementary 1). They could be infected by ToBRFV, but did not show any systemic symptoms. A similar tolerant reaction has been identified in S*.* *lycopersicum* and *S.* *pimpinellifolium* (Ashkenazi et al. 2018; Zinger et al. 2021). However, in addition to the cultivated lines of *S.* *lycopersicum* var. cerasiforme and *S.* *pimpinellifolium*, we also demonstrated tolerance in accessions of the wild tomato plants of *S.* *habrochaites* and *S.* *chilense*.

The reaction of *S. ochranthum*, a close relative to tomato, varied greatly. Two accessions (PI 473,498 and PI 230,519) showed transitional mild systemic mosaic symptoms followed by total recovery on the new apical leaves. While ToBRFV could be detected by bioassays in the mosaic displaying leaves, no virus was present later on the new symptomless top leaves, indicating either the arrest of virus movement or the very strict control of virus replication. Interestingly, similar recovery from disease, including vanishing of symptoms and lack of detectable viruses, has been already reported in *S. ochrantum* when inoculated with the potexvirus, pepino mosaic virus (Soler-Aleixandre et al. 2007).

In contrast to the accessions PI 473,498 and PI 230,519, plants of three *S. ochranthum* accessions (LA2160, LA2162, and LA2166) inoculated with ToBRFV remained symptomless both locally and systemically and the virus could be detected only on the inoculated leaves of these plants. These results proved their high level of resistance to ToBRFV. High resistance of *S. ochranthum* was also found against TMV and ToMV, which suggest the same genetic background of resistance to different tobamoviruses in these plants. Reactions of *S. ochranthum* have been studied so far to cucumber mosaic virus (CMV) and pepino mosaic virus (PepMV) (Rick 1988; Soler-Aleixandre et al. 2007). This is the first paper dealing with the reactions of *S. ochranthum* to tobamoviruses. It would be of special interest to know whether *S. ochrantum* is susceptible or resistant to other important solanaceous pathogenic tobamoviruses like tomato mild mottle virus (ToMMV) or obuda pepper virus (ObPV).

The transfer to cultivated tomato of this high level of resistance of *S. ochrantum* to ToBRV is difficult due to the sexual incompatibility between *S. ochranthum* and *S. lycopersicum* or other closely related tomato species. A potential alternative to surpass this genetic barrier will be the use of somatic hybridization among accessions of these species (Rick 1979; Rick and Chetelat 1995; Pertuzé et al. 2002; Kole 2011).

## Supplementary Information

Below is the link to the electronic supplementary material.

Supplementary file1 (XLSX 52 KB)

## Data Availability

The data that support the findings of this study are available from the corresponding author upon reasonable request.
